# Economic arguments in migrant health policymaking: proposing a research agenda

**DOI:** 10.1186/s12992-020-00642-8

**Published:** 2020-11-20

**Authors:** Nora Gottlieb, Ursula Trummer, Nadav Davidovitch, Allan Krasnik, Sol P. Juárez, Mikael Rostila, Louise Biddle, Kayvan Bozorgmehr

**Affiliations:** 1 Department of Health Care Management, Berlin Technical University, Berlin, Germany; 2 Department of Population Medicine and Health Services Research, School of Public Health, Bielefeld University, Bielefeld, Germany; 3 Center for Health and Migration, Vienna, Austria; 4 Department of Health Systems Management, School of Public Health, Ben-Gurion University of the Negev, Beer Sheva, Israel; 5 Department of Public Health, Center for Migration, Ethnicity and Health, University of Copenhagen, Copenhagen, Denmark; 6 Department of Public Health Sciences, Stockholm University, Stockholm, Sweden; 7 Centre for Health Equity Studies (CHESS), Stockholm University/Karolinska Institute, Stockholm, Sweden; 8 Section for Health Equity Studies and Migration, Department of General Practice and Health Services Research, University Hospital Heidelberg, Heidelberg, Germany

**Keywords:** Discourse analysis, Economics, Equity, Health economics, Health policy, Health political science, Migrant health, Political decision-making, Political economy, Translational research

## Abstract

Welfare states around the world restrict access to public healthcare for some migrant groups. Formal restrictions on migrants’ healthcare access are often justified with economic arguments; for example, as a means to prevent excess costs and safeguard scarce resources. However, existing studies on the economics of migrant health policies suggest that restrictive policies increase rather than decrease costs. This evidence has largely been ignored in migration debates. Amplifying the relationship between welfare state transformations and the production of inequalities, the Covid-19 pandemic may fuel exclusionary rhetoric and politics; or it may serve as an impetus to reconsider the costs that one group’s exclusion from health can entail for all members of society.

The public health community has a responsibility to promote evidence-informed health policies that are ethically and economically sound, and to counter anti-migrant and racial discrimination (whether overt or masked with economic reasoning). Toward this end, we propose a research agenda which includes 1) the generation of a comprehensive body of evidence on economic aspects of migrant health policies, 2) the clarification of the role of economic arguments in migration debates, 3) (self-)critical reflection on the ethics and politics of the production of economic evidence, 4) the introduction of evidence into migrant health policymaking processes, and 5) the endorsement of inter- and transdisciplinary approaches. With the Covid-19 pandemic and surrounding events rendering the suggested research agenda more topical than ever, we invite individuals and groups to join forces toward a (self-)critical examination of economic arguments in migration and health, and in public health generally.

## Background

The Covid-19 pandemic is a powerful illustration that societies can only be as healthy as their weakest members. Yet, welfare states across the globe restrict access to healthcare for certain migrant groups. Some countries exclude migrants with legal status (such as authorized labor migrants) from their public healthcare schemes, offering private, for-profit health services instead. Several European countries limit healthcare provision for asylum-seekers or predicate it on certain conditions such as length of stay or residence in a designated accommodation center. Undocumented migrants’ access to care is similarly tied to preconditions such as minimum duration of stay or proof of identity, destitution or ability to pay for treatment in many states [1]. These restrictions are often justified with economic arguments; for example, as necessary measures to prevent excessive health care utilization and rising costs. Right-wing nationalist parties–such as the Danish People’s Party, the Dutch Freedom Party, the French National Front and more recently the Italian Lega Nord and the German “Alternative for Germany”–have capitalized on these arguments, invoking zero-sum-scenarios which imply that migrants’ needs come at the expense of autochthonous populations [2]. In the context of economic crises and austerity, this makes a convincing case for many voters. What role should Public Health play in the face of rhetoric that exploits the topic of migration to distract from the adverse effects of ongoing welfare state transformations, and that masks xenophobia with economic arguments?

The public health community has long advocated for equitable healthcare provision, basing its main arguments primarily on human rights and ethical principles. To what extent do these concepts resonate with the electorate? Research into public support for immigration policies suggests that migrants are considered least deserving for public benefits across Europe [3]. Their moral claim to welfare is called into question, hinging it, inter alia, on utilitarian considerations such as employability [4]. Hence, economic arguments evidently play a central role in the conceptualizations of migrants’ deservingness, alongside other value- and evidence-based considerations.

Despite its centrality in migration debates, however, much economic reasoning that typically undergirds restrictive policies is not supported by research. But is there enough evidence to make a valid economic case for health equity?

A growing body of evidence indicates that restrictive policies increase rather than decrease costs (see, e.g., [5, 6]). Yet the public debate ignores such findings and they remain relatively overlooked even within the field. Stockpiling activities such as the recent UCL-Lancet Commission Report [7] only marginally touch on this question. Similarly, restrictive immigration policies were shown to compromise migrants’ health [8], but host countries’ responsibilities in this regard are rarely considered.

So, when does evidence matter in migrant health policymaking? What kind of evidence matters? How can we explain the persistent power of empirically unfounded or counterfactual economic arguments when it comes to justifying restrictive policies toward migrant “others”? Many of these fundamental questions concerning the role of economic arguments in migration debates remain under-discussed.

The Covid-19 pandemic adds urgency to these questions, as economic repercussions, anxieties and feelings of resentment create a new breeding ground for populist backlash and welfare chauvinism. At the same time, it offers an opportunity to contextualize migration and health policy issues. First, issues related to financial crises, environmental degradation, global health threats and migration have long been framed in ways that deflect from the larger context and state responsibilities (for example, as sudden and tragic disasters that could neither be anticipated nor prevented). Such framing has lent credence to narrow policy solutions that play into the political economy of migration policies. The tightening of immigration control, for instance, can drive people into the arms of human smuggling networks and create deportable migrant populations in receiving countries who are vulnerable to exploitation. At the same time, such framing has also been obscuring potential structural solutions such as cross-border social contracts or transfer mechanisms for public spending. Yet the pandemic highlights the interlocking political and social forces on global, national and local levels that underlie both welfare state transformations and migration trends (such as neoliberalization, demographic transition, globalization of markets and labor, technological developments). It thus exposes how economic, environmental, migration and global health crises create a “syndemic” of interrelated conditions, grounded in states’ failure to protect and produce essential public goods, such as social security, within and across national borders [9]. Second, the Covid-19 pandemic gives rise to abundant evidence on the intersections between structural vulnerability and health. It could thus serve as an impetus to hold states accountable for the health consequences of their migration policies. Third, migrants are likely to experience an excess risk of Covid-19 related consequences that “might pose an added overall health risk during the pandemic not just for migrants but for all parts of society” [10]. The pandemic thus demonstrates that even affluent countries cannot afford excluding migrants from the social determinants of health. Fourth, however, the Covid-19 pandemic also showcases how limited the impact of evidence on policy decisions can be. The pandemic is an extreme situation that amplifies pre-existing trends and, like a magnifying glass, can improve our understanding of the new “normal”.

## Proposing a research agenda on economic aspects of migrant health policies

The public health community shares societies’ responsibility to counter xenophobia and racism. Toward this ultimate goal we suggest a research agenda on economic arguments in migrant health policymaking, which includes the following five points: 1) the generation of evidence on economic aspects of migrant health policies; 2) the clarification of the role of economic arguments in migration debates; 3) reflection on the ethics and politics of economic evidence production; 4) research for social and policy impact; and 5) the promotion of inter- and transdisciplinarity. The agenda reflects insights from the activities of a dedicated work group, founded 2016 under EUPHA’s Migrant and Ethnic Minority Health Section, and from a workshop at the 2019 European Public Health Conference. With an eye to the effects of the Covid-19 outbreak on the socio-economic conditions and health of migrants, as well as the political dynamics surrounding the pandemic, we deem the suggested agenda more urgent than ever.

### Generating evidence on economic aspects of migrant health policies

The first goal is the generation of a comprehensive body of evidence on economic aspects of policies affecting migrants’ health. This goal requires a systematic assessment to find out which policies relate to migrant health and how (for example, in the sense of evaluating coherences and contradictions within national policy frameworks). It also includes mapping existing information and developing solutions for methodological challenges. The availability of quantitative data on migrant health must be improved through inclusion of migrants in routine health monitoring and tailored data collection. Untapped data sources should be explored; for example, through collaborations with civil society organizations. The yearly report of the International Observatory on Access to Healthcare by the non-governmental organization Médecins du Monde (https://mdmeuroblog.wordpress.com/resources/publications/) serves as a positive example of how humanitarian and political work with migrants can produce a wealth of information that is not captured by health information systems*.* Qualitative methods can help illuminate intangible aspects that remain hidden in classic economic analysis, as demonstrated by the micro-costing methodology of the EquiHealth study. The study drew on quantitative and qualitative data (such as information on living and working conditions and health-seeking behaviours, including informal care) to construct migrant healthcare vignettes, modelling patient trajectories and the costs of different healthcare access scenarios [6]. Such approaches can shed light on how divergent migrant health policies “play out” during the Covid-19 pandemic in terms of health and socio-economic outcomes. They can also be used to examine how changing public policies affect the health and social conditions for various migrant groups (for example, labor migrants, asylum-seekers and refugees, undocumented migrants, unaccompanied minors, and persons who migrated for the purpose of family unification) in the Covid-19 aftermath.

### Clarifying the role of economic arguments in migration debates

The second goal relates to the scrutiny of economic arguments in migration debates. A better understanding of how different arguments and contextual dynamics interact and influence policymaking is required. Explorative studies on concepts of “health-related deservingness” can serve as a starting point (see, e.g., [11]). Future research should help explain why some economic arguments “catch on”–despite opposing evidence–while others go unheard. Acknowledging that “migrants” are a heterogenous groups with differential entitlements and positions in policy and discourse, research should make explicit the differing cost-benefit analyses, implicit willingness-to-pay thresholds and varying equity considerations made by policymakers for different migrant and non-migrant groups. This goal further includes a critical analysis of the political economy of migration, and of narratives illuminating or obscuring the roles of different actors (migrants, host communities/states, sending communities/states, employers, recruitment agencies, etc.). For example, who are the profiteers, the payers, and the losers from migration processes? Covid-19-related border closures and their repercussions on different population groups–including, for example, acute crises in the delivery of elderly care in high-income countries–can serve as useful examples to study the counterfactuals of a world with no (or substantially reduced) cross-border migration that seemed impossible in the pre-pandemic era. This, in turn, may help generate evidence on the contributions of migrants to host communities and frame social and health service provision as an investment that reaps benefits for receiving communities and migrants alike in the long term.

### Reflecting on the ethics and politics of economic evidence production

We propose (self-)critical reflection on the ethics and politics of the production of economic evidence as a third goal. Despite claims to scientific objectivity, researchers’ choice of terms and models is entrenched in certain paradigms. It is never “neutral” or “objective”. For example, even well-intended arguments–for instance, that migrants contribute to host economies–must be well reflected as they link the value of humans to their economic utility. Furthermore, economic models often embed ethical norms as basic assumptions [12]. For example, the claim that restricted access to primary healthcare ultimately entails higher costs presupposes that urgent treatment is provided unconditionally (for instance, as a matter of medical ethics). To what extent should economic assessments make such assumptions explicit?

As another example, we propose reflecting on what it means to focus on “migrant health” within the wider socio-political context. How can researchers, activists and policymakers avoid reproducing the very dichotomy–“us” versus “them”–they want to overcome? How can these actors avoid misleading simplifications (for example, migrants as one homogenous group) and essentialisms (for example, migrant women as victims)? How do they prevent reaffirming other groups’ feeling of being “left behind” (the very feeling that may make them susceptible to populism)? How can health rather be used to establish common ground between different groups?

We suggest two interrelated approaches to resolve these contradictions: First, we propose integrating migration in the wider context of societies’ responses to increasing diversity. Taking an intersectional approach, different formal migration categories should be treated as determinants of potential disadvantage or privilege alongside other aspects such as socio-economic status, gender identity, and sexual orientation. Second, in line with an intersectional approach, we recommend focusing more research on the upstream factors that shape the health of both migrant and non-migrant populations. In doing so, research can inform health interventions that are relevant and beneficial for migrant communities and society as a whole.

### Doing research for social and policy impact

The fourth goal is to foster evidence-informed political debates on migrant health. Knowledge on science communication exists and the Covid-19 pandemic has underscored its importance. We propose that researchers in the migration and health realm tap more into this resource to communicate their study results to different audiences, and to actively introduce them into policymaking processes. We further call on academic institutions to provide learning opportunities and support for science communication, and to incentivize activities aimed at social impact.

However, it must be acknowledged that the production and dissemination of scientific knowledge is also subject to power dynamics. What kind of research is successful in the competition for funding? What kind of research gets published? Which institutions are considered reliable sources of evidence; globally, but also on national and local levels? And, last but not least, who is left out of evidence production and communication? We call on migration and health researchers to problematize and address these questions in their own academic practice as researchers, reviewers and mentors (for instance, by doing “research with” rather than “research about”), and as a community, leveraging collective resources to challenge systemic flaws. Existing initiatives focusing on gender equality in academia or on reforming the academic publishing industry can serve as role models and potential allies.

### Promoting inter- and transdisciplinary research

Finally, we propose adopting inter- and transdisciplinary approaches while working towards these goals. Capitalizing on thematic and methodological intersections between different sectors and disciplines will be key to taking research efforts forward. Untapped options for methodological transfer, synthesis and development can help ameliorate the limited availability and quality of data. Different disciplinary perspectives can facilitate critical inquiry into the entanglement of the factual, ethical and political in the production of economic evidence. Partnerships between academia and practice stakeholders–including migrant communities–can help bridge evidence-policy gaps. These partnerships can both diversify the types of knowledge considered valid in policy debates and strengthen research teams’ commitment to their practice partners’ ultimate goal: the translation of evidence into positive change on the ground.

## Conclusions

The public health community has a responsibility to counteract xenophobia and racism. In debates on migrants’ health rights, restrictive policies are often justified with economic arguments. Yet, evidence to substantiate these arguments is lacking. To foster transparent debates on migrant health policies–including the debunking of anti-migrant rhetoric–we propose a research agenda that comprises five goals: the generation of evidence on the economics of migration and health, scrutiny of the role of economic arguments in migrant health rights debates, reflection on economic evidence production, science communication, and the endorsement of inter- and transdisciplinarity. Our research agenda stakes out a wide field that can be worked from various angles. Our work group warmly welcomes individuals and groups who embrace the professional challenges and political vision to join forces toward the promotion of a (self-)critical examination of economic arguments in migration and health during the Covid-19 pandemic and beyond.

## Data Availability

Not applicable.
